# Sustained weight loss exceeding 100 kg with sequential incretin-based therapy in Prader–Willi syndrome

**DOI:** 10.1210/jcemcr/luag260

**Published:** 2026-09-16

**Authors:** Marlon Yovera-Aldana, Helard Manrique-Hurtado, Guillermo E Umpierrez

**Affiliations:** Grupo de Investigación de Neurociencias, Metabolismo, Efectividad Clínica y Sanitaria, Universidad Científica del Sur, Lima 15816, Perú; Deparment of Endocrinology, Clínica Delgado—AUNA, Lima 15074, Perú; Division of Endocrinology, Department of Medicine, Emory University School of Medicine, Atlanta, GA 30322, USA

**Keywords:** Prader–Willi syndrome, retatrutide, incretin-based therapy, obesity, type 2 diabetes remission, hypogonadotropic hypogonadism

## Abstract

Prader–Willi syndrome (PWS) is a neurogenetic disorder characterized by hyperphagia, severe obesity, and early-onset metabolic complications, in which durable weight loss is rarely achieved. We report a 29-year-old man with genetically confirmed maternal uniparental disomy PWS who presented with severe obesity (185 kg; body mass index [BMI] 59.7 kg/m^2^) and newly diagnosed type 2 diabetes (glycated hemoglobin [HbA1c], 8.3% [SI: 67 mmol/mol] [reference range, <5.7% (SI: <39 mmol/mol)]). He received sequential incretin-based therapy, initiated with retatrutide (1-12 mg weekly) within a clinical trial, followed by oral semaglutide and then dulaglutide after retatrutide became unavailable, together with a structured hypocaloric diet and supervised exercise. At 18 months, total weight loss reached 58.8% (−108.7 kg), with HbA1c of 4.9% (SI: 30 mmol/mol), consistent with diabetes remission after discontinuation of all glucose-lowering medications. Mild transient gastrointestinal symptoms occurred during dose escalation, with no serious adverse events. Follow-up bioimpedance showed a skeletal muscle mass of 38.5 kg after a 58.8% reduction in total body weight.

## Introduction

Prader–Willi syndrome (PWS) is a complex neurogenetic disorder caused by the loss of expression of paternally inherited genes on chromosome 15q11–q13. Approximately 20% to 30% of cases result from maternal uniparental disomy of chromosome 15, whereas most cases are caused by paternal deletions of the same region [1]. It is characterized by severe hypothalamic dysfunction leading to hyperphagia, hypogonadism, and severe obesity [1, 2]. Obesity is the principal determinant of morbidity, and reduced life expectancy, with mortality occurring in the fourth decade due to obesity-related complications [3].

Management of obesity in PWS is exceptionally challenging. Conventional strategies, including environmental food control and behavioral interventions, are difficult to sustain [4]. Pharmacologic options have historically shown limited and heterogeneous efficacy; for instance, although case reports of glucagon-like peptide-1 receptor agonists (GLP-1 RAs) appeared promising, recent meta-analyses of clinical trials have shown no clinically significant weight reduction in patients with PWS, despite some improvements in satiety [5, 6]. Other therapies, such as diazoxide choline, primarily target hyperphagia behaviors without substantial effects on body mass [7].

The emergence of multireceptor agonists represents a new frontier. Retatrutide is a novel triple agonist targeting the GLP-1, glucose-dependent insulinotropic polypeptide (GIP), and glucagon receptors [8]. By combining appetite suppression with increased energy expenditure through glucagon receptor activation, retatrutide has achieved mean weight losses exceeding 24% in phase 2 trials of nonsyndromic obesity [9]. However, individuals with PWS have been systematically excluded from these landmark trials, and the role of such intensive incretin-based therapy in this population remains unknown [10].

Herein, we report the case of a functional adult with genetically confirmed PWS who achieved an exceptional and sustained weight loss exceeding 100 kg, along with complete remission of type 2 diabetes, after a strategic, sequential incretin-based therapy combined with a structured lifestyle intervention.

## Case presentation

A 29-year-old man with a known diagnosis of PWS due to maternal uniparental disomy (mUPD) (15q11–q13), confirmed by methylation-specific polymerase chain reaction and multiplex ligation-dependent probe amplification, presented in December 2023 for evaluation of severe obesity and hyperglycemia.

He was born at term via cesarean section after an uncomplicated pregnancy. During infancy, he exhibited generalized hypotonia, feeding difficulties, and delayed motor milestones. By the age of 3 years, he developed hyperphagia and progressive weight gain. Throughout childhood, he continued to have reduced muscle tone and excessive daytime sleepiness.

His past medical history included surgical repair of left-foot syndactyly at age 5 and bilateral knee surgeries at age 17 for valgus deformities related to excessive body weight. He also had lumbar scoliosis and pectus carinatum without respiratory compromise. Puberty did not occur spontaneously.

Despite these features, the patient achieved a high level of functional independence. Neuropsychological evaluation revealed preserved executive function, intact reasoning abilities, and normal verbal comprehension. He completed secondary education and obtained a technical diploma, maintaining full-time employment and independent living. Behavioral assessment showed mild rigidity and anxiety related to food restriction, without features suggestive of autism spectrum disorder or psychosis. He demonstrated strong adherence to structured routines and high motivation for treatment.

At presentation, body weight was 185 kg, corresponding to a body mass index (BMI) of 59.7 kg/m^2^. Blood pressure was 128/82 mmHg. Physical examination revealed generalized obesity, reduced muscle tone, small testes, and sparse secondary sexual characteristics, without cushingoid features.

## Diagnostic assessment

At presentation, laboratory findings confirmed newly diagnosed type 2 diabetes mellitus, with elevated fasting plasma glucose and glycated hemoglobin (HbA1c) levels (Table 1). Endocrine evaluation revealed hypogonadotropic hypogonadism, with low testosterone, luteinizing hormone (LH), and follicle-stimulating hormone (FSH) levels (Table 1). Thyroid function was normal, and morning cortisol concentration was within the reference range. Insulin-like growth factor 1 (IGF-1) was within the lower-normal range for age and sex. The patient had no documented history of growth hormone therapy, and adult growth hormone deficiency was not formally evaluated by stimulation test. An IGF-1 standard deviation score was not available because it was not provided by the laboratory and could not be retrospectively calculated using assay-specific reference data. Brain magnetic resonance imaging demonstrated mild hypothalamic atrophy with preserved pituitary anatomy.

**Table 1:** Metabolic and hormonal parameters during treatment and follow-up

Parameter	Baseline (Dec 2023)	18 months (Jun 2025)	Reference range
Fasting glucose	122 mg/dL (6.8 mmol/L)	84 mg/dL (4.7 mmol/L)	70-100 mg/dL (3.9-5.6 mmol/L)
HbA1c	8.3% (67 mmol/mol)	4.9% (30 mmol/mol)	<5.7% (<39 mmol/mol)
Triglycerides	230 mg/dL (2.6 mmol/L)	102 mg/dL (1.2 mmol/L)	<150 mg/dL (<1.7 mmol/L)
HDL cholesterol	34 mg/dL (0.88 mmol/L)	52 mg/dL (1.35 mmol/L)	>40 mg/dL (>1.0 mmol/L)
ALT	78 U/L (1.3 µkat/L)	29 U/L (0.48 µkat/L)	<45 U/L (<0.75 µkat/L)
Creatinine	0.9 mg/dL (80 µmol/L)	0.8 mg/dL (71 µmol/L)	0.7-1.3 mg/dL (62-115 µmol/L)
Testosterone	30 ng/dL (1.0 nmol/L)	800 ng/dL (27.7 nmol/L)	300-1000 ng/dL (10-35 nmol/L)
LH	0.5 IU/L	4.6 IU/L	1.5-9.0 IU/L
FSH	0.7 IU/L	5.1 IU/L	1.0-12.0 IU/L
TSH	2.1 µIU/mL (2.1 mIU/L)	2.0 µIU/mL (2.0 mIU/L)	0.4-4.0 mIU/L
IGF-1	118 ng/mL (15.4 nmol/L)	242 ng/mL (31.7 nmol/L)	100-300 ng/mL (13-39 nmol/L)
Morning cortisol	13.5 µg/dL (373 nmol/L)	—	5-25 µg/dL (138-690 nmol/L)

Abbreviations: ALT, alanine aminotransferase; FSH, follicle-stimulating hormone; HDL, high-density lipoprotein; IGF-1, insulin-like growth factor 1; LH, luteinizing hormone; TSH, thyroid-stimulating hormone.

## Treatment

Weight management was initiated in December 2023 through participation in a registered clinical trial evaluating retatrutide for severe obesity and metabolic disease. According to the patient, written informed consent was obtained before enrollment and protocol-specific screening procedures were completed. Dosing began at 1 mg once weekly and was gradually titrated to 12 mg once weekly per the study protocol.

Concurrently, lifestyle interventions were implemented under local supervision. Per the patient's report, these included a hypocaloric dietary program, adherence to a vegan diet during the first three months, and progressive increases in physical activity. Physical activity consisted primarily of walking between December 2023 and March 2024, followed by more intensive cardiovascular exercise thereafter.

After completing the 8-month trial, the patient relocated internationally for work-related reasons and was unable to continue receiving retatrutide. To maintain the metabolic benefits observed during the trial, oral semaglutide (7 mg once daily) was recommended based on local availability and accessibility. During subsequent endocrinology follow-up, treatment was transitioned to dulaglutide (1.5 mg once weekly) due to medication availability and access considerations.

All glucose-lowering medications were discontinued in March 2025 after sustained normalization of glycemic parameters.

To address persistent hypogonadotropic hypogonadism, pulsatile gonadotropin-releasing hormone (GnRH) therapy was initiated in April 2025 with a portable infusion pump, after which testosterone and gonadotropin levels improved.

## Outcome and follow-up

**Figure 1:**
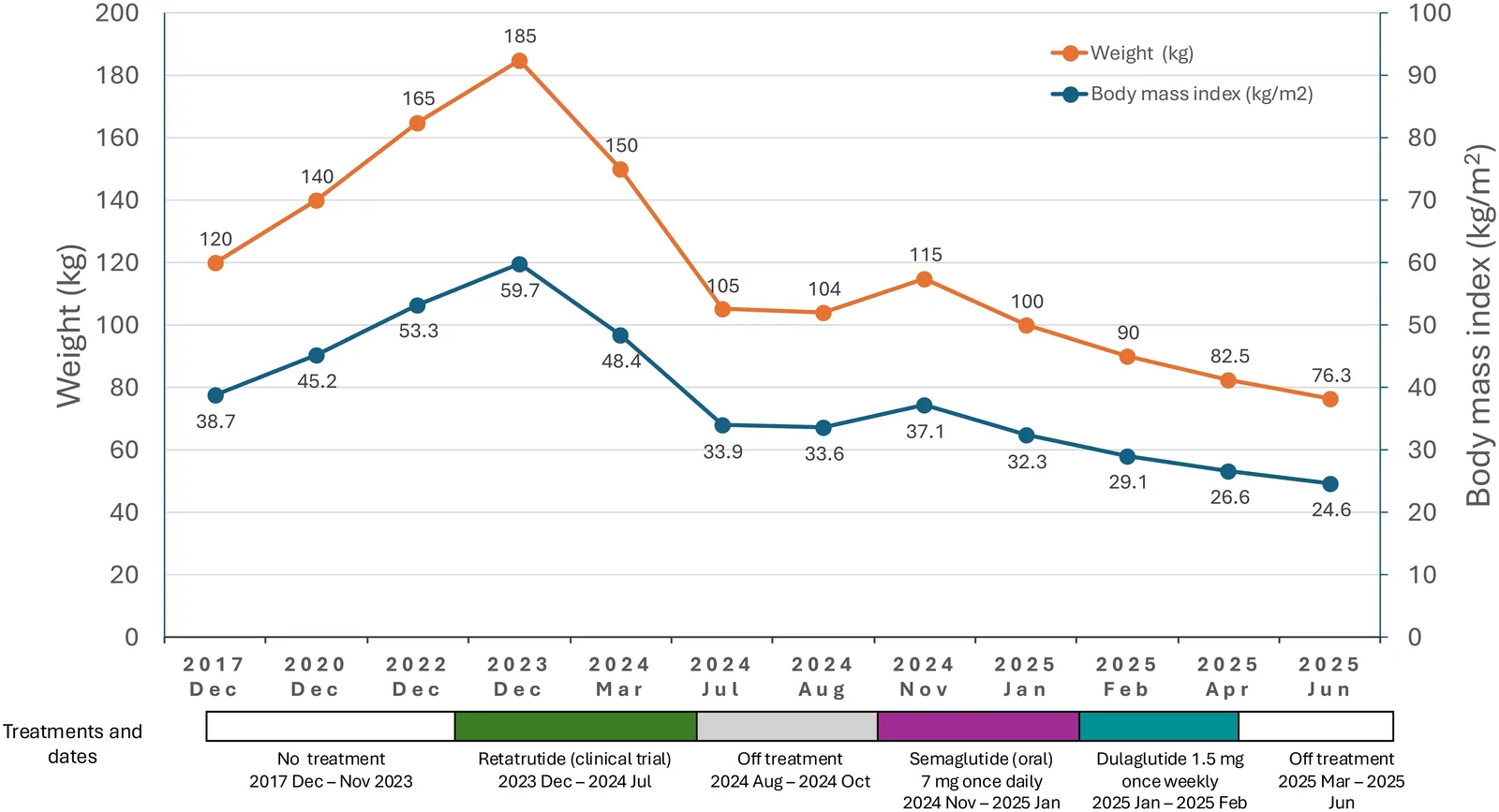
Longitudinal changes in body weight and BMI from 2017 to 2025. Weight (kg) and BMI (kg/m^2^) are shown over time. Sequential treatment periods with retatrutide, semaglutide, and dulaglutide are indicated. Marked weight reduction occurred during retatrutide therapy and was maintained throughout follow-up.

After 8 months of retatrutide therapy, body weight decreased from 185 to 105 kg—a 43% reduction in total body weight that lowered the BMI from 59.7 to 33.8 kg/m^2^ (Fig. 1). Glycemic control also improved markedly, with HbA1c falling from 8.3% (SI: 67 mmol/mol) to 4.9% (SI: 30 mmol/mol) (Table 1).

Weight loss continued after transitioning to oral semaglutide (7 mg daily) and then dulaglutide (1.5 mg weekly), while metabolic parameters remained stable. At the 18-month follow-up from initiation of retatrutide therapy (and approximately 4 months after discontinuation of dulaglutide), body weight had decreased from 185.0 to 76.3 kg, representing a total weight loss of 108.7 kg (58.8% of initial body weight). Glycemic control remained excellent, with glycated hemoglobin (HbA1c) declining from 8.3% (SI: 67 mmol/mol) to 4.9% (SI: 30 mmol/mol) and fasting plasma glucose from 122 mg/dL (SI: 6.8 mmol/L) to 84 mg/dL (SI: 4.7 mmol/L), consistent with sustained diabetes remission during sequential incretin-based therapy.

Follow-up multifrequency bioimpedance analysis observed a fat-free mass of 64.1 kg and skeletal muscle mass of 38.5 kg, while triglycerides and alanine aminotransferase levels normalized.

No episodes of clinically significant hypoglycemia or serious adverse events were reported during follow-up. Mild transient gastrointestinal symptoms occurred during dose escalation and did not require discontinuation of treatment.

Following initiation of pulsatile GnRH therapy, serum testosterone increased from 30 ng/dL (SI: 1.0 nmol/L) to 800 ng/dL (SI: 27.7 nmol/L), accompanied by normalization of LH and FSH levels within 10 weeks. The patient also reported improvements in energy, physical performance, and libido.

At the last follow-up, the patient remained euglycemic, normotensive, physically active, fully independent, and weight stable.

## Discussion

We report a case of an adult with genetically confirmed PWS who achieved extreme and sustained weight loss exceeding 50% of total body weight, along with complete remission of type 2 diabetes, following sequential incretin-based therapy. To our knowledge, this represents one of the largest and most durable weight reductions reported in PWS without bariatric surgery.

Management of obesity in PWS remains highly challenging due to the complex interplay of hypothalamic dysfunction and hyperphagia [11]. While multidisciplinary approaches remain the cornerstone, their long-term efficacy is often limited by physiological adaptations that favor weight regain [12]. Conventional pharmacological options have historically yielded disappointing results; for instance, while GLP-1 receptor agonists like liraglutide show benefits in satiety, their impact on BMI in controlled trials of PWS patients has been inconsistent [13, 14]. More recently, a case series and literature review by Koceva et al [15] reported that long-term semaglutide therapy was associated with preventing further weight gain or achieving weight reductions of 11% to 14.4% in adults with PWS, supporting a potential role for incretin-based therapies in this population. Other emerging therapies, such as diazoxide choline, focus on hyperphagia-related behaviors but have shown only modest effects on overall adiposity [7, 16]. Furthermore, while bariatric surgery is an option for selected cases, its long-term safety and success in this population remain under scrutiny due to the risk of severe complications and weight relapse [11, 17].

In this context, the magnitude of response observed in our patient is exceptional. Retatrutide, a triple-hormone-receptor agonist (GLP-1, GIP, and glucagon), has demonstrated mean weight losses exceeding 24% in phase 2 trials for nonsyndromic obesity [10]. The 59% total body weight substantially exceeds the weight losses reported with semaglutide in the currently available PWS literature [14]. This suggests that targeting multiple metabolic pathways—suppressing appetite via GLP-1/GIP and increasing energy expenditure via glucagon receptors—may be particularly effective for the severe metabolic derangements of PWS [8].

Genotype may have significantly influenced this outcome. Individuals with mUPD, like our patient, often exhibit milder maladaptive behaviors and better executive functioning compared to those with paternal deletions. These phenotypic characteristics likely facilitated the high level of adherence to the structured dietary and exercise interventions that were maintained even after the cessation of triple-agonist therapy [18, 19].

The generalizability of this case should be interpreted with caution. Our patient had mUPD, preserved executive functioning, independent living, and a high degree of treatment adherence. These characteristics may not be representative of many adults with PWS, particularly those with paternal deletions, greater cognitive impairment, more severe behavioral manifestations, or those living in institutional settings where environmental and behavioral factors differ substantially. Therefore, similar outcomes should not be assumed across the broader PWS population [20, 21].

Additionally, the recovery of the hypothalamic–pituitary–gonadal axis in our patient is a remarkable finding. Hypogonadism in PWS is typically multifaceted, involving both central and peripheral defects [22, 23]. The marked improvement in testosterone and gonadotropin levels observed after substantial weight loss and subsequent pulsatile GnRH therapy suggests that hypothalamic–pituitary–gonadal function remained at least partially responsive. However, the relative contributions of weight reduction, incretin-based therapy, and pulsatile GnRH treatment cannot be determined from this single case [24].

A limitation of this report is the relatively short follow-up after discontinuation of incretin-based therapy. Although substantial weight loss was maintained over 18 months of sequential treatment, longer post-treatment follow-up is needed as weight regain has been reported after withdrawal of GLP-1 receptor agonists in larger studies [25, 26]. Body-composition assessment was limited by the absence of baseline measurements and confirmatory techniques such as dual-energy X-ray absorptiometry. Therefore, changes in lean mass could not be formally quantified, and bioimpedance-derived estimates should be interpreted with caution in the context of severe obesity and major weight loss [27]. In addition, adult growth hormone deficiency was not formally evaluated with a stimulation test, which may limit interpretation of the observed body-composition findings [28].

In conclusion, this case demonstrates that profound weight loss and metabolic remission are achievable in selected adults with PWS using a sequential incretin-based approach. These findings highlight the urgent need for clinical trials investigating triple-agonist therapies specifically within the PWS population.

## Learning points

- Sequential incretin-based therapy, including initial treatment with a triple agonist, may achieve substantial and sustained weight loss in selected adults with Prader–Willi syndrome.
- Sequential incretin-based therapy initiated with retatrutide may produce greater weight reduction than previously reported pharmacologic therapies in syndromic obesity, although evidence remains limited.
- Sustained metabolic remission, including normalization of glycemia after treatment discontinuation, may be achievable when pharmacologic therapy is combined with sustained lifestyle modifications and high treatment adherence.
- Clinical phenotype, including preserved executive function and autonomy, and the ability to maintain dietary and physical activity interventions, may influence treatment response and long-term outcomes in Prader–Willi syndrome.
- Recovery of gonadal function may occur in selected cases with preserved hypothalamic function.

## Contributors

All authors made individual contributions to authorship. M.Y.-A. was involved in the diagnosis, clinical management, data interpretation, and manuscript drafting. H.M.-H. contributed to patient management and data acquisition. G.E.U. contributed to data interpretation and critical revision of the manuscript. All authors reviewed and approved the final draft.

## Data Availability

Original data generated and analyzed for this case report are included in this published article.
